# A memetic algorithm for finding multiple subgraphs that optimally cover an input network

**DOI:** 10.1371/journal.pone.0280506

**Published:** 2023-01-20

**Authors:** Xiaochen He, Yang Wang, Haifeng Du, Marcus W. Feldman

**Affiliations:** 1 Center for Administration and Complexity Science of Xi’an Jiaotong University, Xi’an, Shaanxi Province, China; 2 Morrison Institute for Population and Resource Studies, Stanford University, Stanford, CA, United States of America; Università degli Studi di Bari Aldo Moro: Universita degli Studi di Bari Aldo Moro, ITALY

## Abstract

Finding dense subgraphs is a central problem in graph mining, with a variety of real-world application domains including biological analysis, financial market evaluation, and sociological surveys. While a series of studies have been devoted to finding subgraphs with maximum density, the problem of finding multiple subgraphs that best cover an input network has not been systematically explored. The present study discusses a variant of the densest subgraph problem and presents a mathematical model for optimizing the total coverage of an input network by extracting multiple subgraphs. A memetic algorithm that maximizes coverage is proposed and shown to be both effective and efficient. The method is applied to real-world networks. The empirical meaning of the optimal sampling method is discussed.

## 1. Introduction

Over the past several decades there has been substantial interest in studying social networks beyond the traditional social sciences while maintaining a focus on social structures. Specifically, instead of focusing on demographic attributes of a certain population, an increasing number of studies have focused on the structure of relationships that connect individual behaviors with collective dynamics [1]. One focus of the analysis of network structure has concerned cohesive subgraphs [2]. Notable examples of this work are sociometric cliques [3] and variants such as *n*-cliques, *n*-clan, *k*-plex, or *k*-core [4]. Related work has focused on detecting core/periphery structures [5], rich clubs [6] or communities [7]. Generally, the aim of these studies has been to find one or more subgraphs that maximizes some notion of density.

One popular notion of density that has been widely explored in the literature is the average degree (measured by edge-to-vertex ratio), and the problem of finding a subgraph that maximizes the average degree is called the densest subgraph problem (DSP) [8]. Analysis of the DSP has been applied to DNA analysis [9, 10], financial market evaluation [11], social surveys [12, 13], and theoretical computer science [14, 15]. In the Web domain, Gibson et al. identified the link spams by extracting dense subgraphs in large graphs [16], which is one of the greatest challenges in evaluating search engine rankings [17]. In the social context, DSP was applied to expert team formation [15, 18] as well as party organization [19, 20]. Angel et al. detected real-time stories by searching for dense subgraphs in the entity co-occurrence graph constructed from micro-blogging streams [21]. DSP has been also employed to find teams with higher collaborative compatibility [22].

DSP aims at extracting a single subgraph, but many real-world cases seek a collection of dense subgraphs, such as communities or social stories [23]. There are relatively few studies in this direction, one of which, by Balalau et al., focused on finding a set of *m* subgraphs that maximizes the total density of each subgraph (denoted as the “multiple-*m* densest subgraphs problem”, M*m*DSP) [23]. Variants of this model have been proposed subsequently [24, 25]. These studies have solved the problem of how to extract multiple dense subgraphs, but the process of covering the input network by extracting multiple subgraphs has not been addressed. Maximizing the subgraph density and maximizing the covering have different social meanings. In many real-world cases, the density of subgraphs does not have to be large. For example, a collection of network surveys may not focus on how dense each investigated network is, but on how best to cover the whole population, which is a boundary specification problem [26, 27]. In a network survey, self-report of social relationships is commonly used to collect network data. Specifically, given a list of participants, the data are obtained from answers to single-item questions that ask participants to enumerate individuals to whom they are connected by a direct relationship of a specified kind [1, 28]. The main purpose of such a network survey is to best cover the interactional relationships. Besides network surveys, the covering problem can be also applied to influence maximization [29], network tomography [30], or pinning control [31].

**Fig 1:**
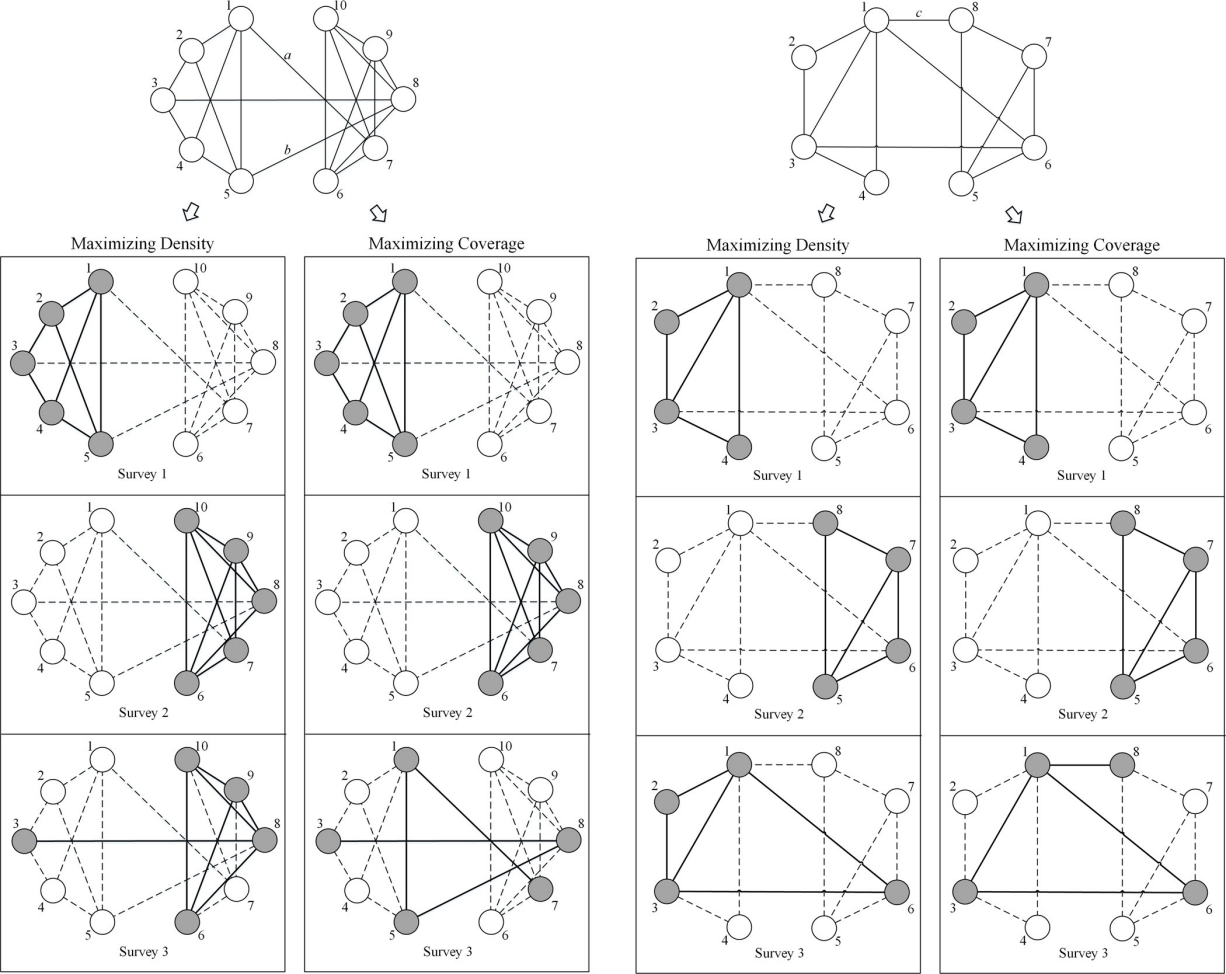
Two illustrations of the difference between maximizing the density and the coverage. The upper two networks are the input networks. The lower part contains the solution of extracting multiple subgraphs. In the solution part, grey nodes represent the included nodes and white nodes represent the omitted nodes. The dashed lines represent the unobserved ties, while the bold solid lines represent the extracted ties.

The present study addresses the problem of how to find multiple subgraphs that best cover the input network. We call the problem “multiple-*m* covering *k*-subgraphs problem” (M*m*C*k*SP), i.e., maximizing the covering of the network edges given *m* subgraphs of limited size *k*. Unlike the classic graph partitioning problem and the densest subgraph problem, the present study aims to find how to multiply extract subgraphs that leads to the best coverage of the network relationships. Two illustrations that show the difference between M*m*C*k*SP and the densest subgraph problem are in Fig 1. Given the input network in Fig 1A, standard community detection finds the partitions of {1,2,3,4,5} and {6,7,8,9,10}. If we set the number of subgraphs to 3 and the subgraph size to 5, M*m*DSP may present the best solution as the partitions of {1,2,3,4,5}, {6,7,8,9,10} and {3,6,8,9,10} in order to maximize the density of each extracted subgraph, while M*m*C*k*SP may extract the subgraphs {1,2,3,4,5}, {6,7,8,9,10} and {1,3,5,7,8}, which may cover all the network edges even though the subgraph {1,3,5,7,8} is not dense. M*m*DSP and M*m*C*k*SP also extract different subgraphs in Fig 1B. Here the edges *a*, *b* in (a) and *c* in (b) are ignored using M*m*DSP, but these omitted edges connecting different communities may sometimes have a useful social interpretation. Covering these edges can help provide a better understanding of the input network structure.

**Fig 2:**
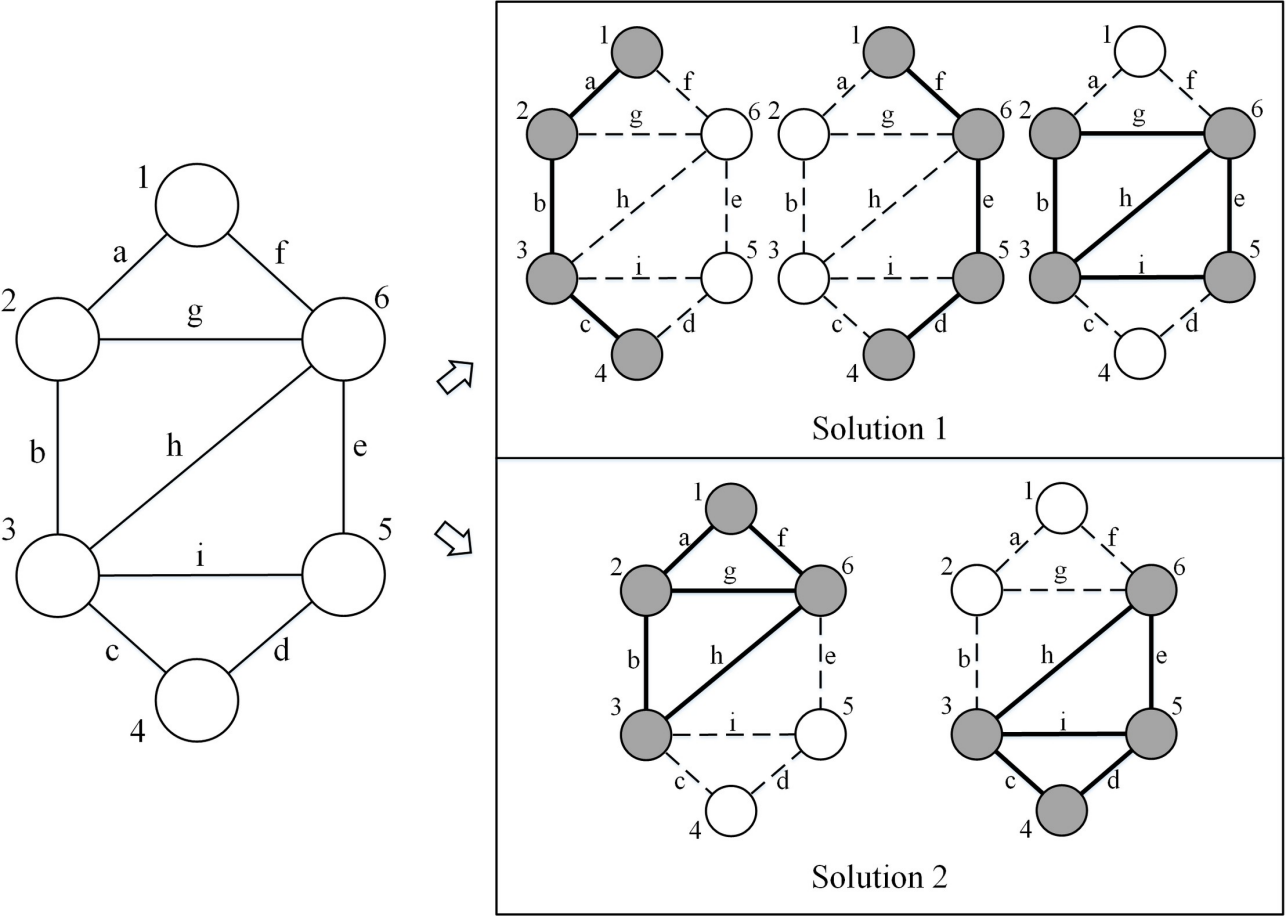
An illustration of the optimization problem of finding multiple subgraphs that best cover the input networks. The left part is the input network which consists of six nodes and nine edges. The right part contains the solutions by extracting multiple subgraphs. Solution 1 requires three subgraphs and solution 2 requires two subgraphs. In the solution part, grey nodes represent the included nodes and white nodes represent the omitted nodes. Dashed lines represent unobserved ties, while bold solid lines represent the extracted ties.

In real world cases, the subgraph size and the number of subgraphs should be constrained because they are always associated with costs. Taking network surveys as an example, a larger nominalist of nodes makes the burden on respondents greater, in which case ties are more likely to be missed because respondents may not be able to recall enough to fully capture the network structure [32]. Here, we formalize M*m*C*k*SP as a new optimization problem, which goes beyond the conventional strategy of optimizing network density. An illustration of the optimization is shown in Fig 2. Given an input network consisting of six nodes and nine edges, if we constrain the subgraph size to 4, we can extract subgraphs {1, 2, 3, 4}, {1, 4, 5, 6} and {2, 3, 5, 6} that cover all ties in the entire population. Solution 2 extracts {1, 2, 3, 6} and {3, 4, 5, 6}, which can also include all the edges. Obviously, solution 2 in Fig 2 is more cost-effective than solution 1. Here, we design an algorithm that can find the most cost-effective solution.

The present study is organized as follows: related background, including the densest subgraph problem, and corresponding strategies for the problem with multiple subgraphs, including optimization models, are presented in section 2. In section 3, we propose a memetic algorithm that optimizes the covering problem for each subgraph. Experiments with the proposed algorithm on computer-generated and real-world networks are described in section 4. Section 5 presents the conclusion and discussion.

## 2. Background

### 2.1. The densest subgraph problem and the solution approach

The densest subgraph problem (DSP) refers to how to obtain a list of members with the highest density. Given a graph *G*(*V*, *E*), where {*v*_*i*_} ∈ *V* denotes the set of nodes and {*e*_*ij*_} ∈ *E* denotes the set of relationships, DSP aims to find a subgraph *G*’(*V*’, *E*’) whose average density of *G’* computed as $\frac{\left|E'\right|}{\left|V'\right|}$ is the largest [8]. The optimization of DSP can then be formulated as (1), below. Solution of the DSP has been shown to require polynomial time [8, 33–35].


$$\text{Maximize}\;\frac{\left|E'\right|}{\left|V'\right|},\;\text{s}.\text{t}.\;V'\subseteq V.$$ (1)

The average density of the extracted subgraph in DSP is associated with the subgraph size, and there is a tradeoff between the density and size [36]. From DSP, one may extract smaller subgraphs in sparser networks but extract larger subgraphs in denser networks. However, in real applications there always exists an upper bound for the subgraph, and one may constrain the size of dense subgraphs [36, 37]. If all subgraphs have the same (bounded) size, the problem, which then becomes NP-hard [14, 33], has been investigated under various names including the “*k*-cluster problem” [38–40], the “*k*-cardinality subgraph problem” [41], or the “densest *k*-subgraph problem” (D*k*SP) [42, 43]. This problem is formulated as (2), below. Some variants of D*k*SP has been proposed. If the extracted subgraph is required to be connected, the problem is referred as to the densest connected *k*-subgraph problem (DC*k*SP) [44]. In weighted networks, finding the subgraph with *k* nodes that has the highest sum of the weights (edges) is called the “heaviest k-subgraph problem” (H*k*SP) [45]:

$$\text{Maximize}\;\frac{\left|E'\right|}{\left|V'\right|},\;\text{s}.\text{t}.\left|V'\right|\leq k\leq \left|V\right|$$ (2)

D*k*SP actually has important interpretations in social science. A social problem related to D*k*SP is called the “boundary specification problem” (BSP), which aims to find a list of samples that best represents the population [26]. When nodes are excluded from the system, the observed network structure differs from the actual one. Simulations have examined features of missing actors and have shown the detrimental impact of incomplete sampling [27, 28, 46, 47]. The similarity between the sample and the complete network declines as more nodes are excluded, and missing nodes substantially affect measures related to the complete network [28, 46, 47].

**Fig 3:**
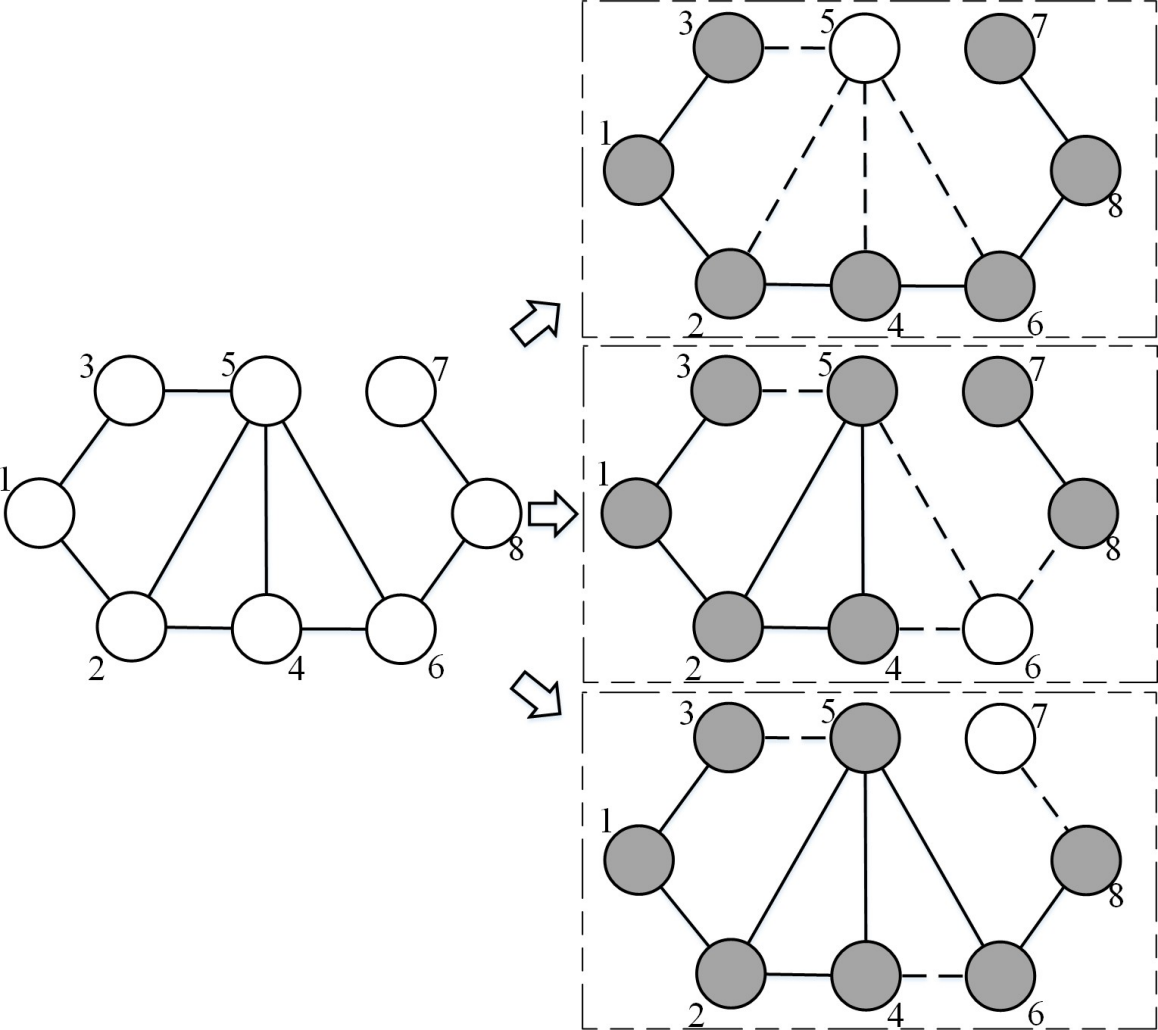
DkSP in solving the boundary specification problem. Grey nodes represent the included nodes and white nodes represent the omitted nodes. The dashed lines represent unobserved ties. The left network is the input network, while the three networks on the right represent the solution made up of extracted subgraphs. The upper right network excludes node 5 and becomes a ring; the middle right network excludes node 6 and becomes unconnected; the lower right network excludes node 7, and is the best solution of D*k*SP.

Solving D*k*SP can help to solve BSP, as illustrated in Fig 3. The input network consists of eight nodes, and we set the subgraph size *k* at 7. If we exclude node 5, then the network has a ring structure, which is quite different from the original structure. If node 6 is omitted by accident or for convenience, the whole network becomes unconnected. This example illustrates how a minor change in network structure can have a dramatic effect on inference about network properties as a whole [48]. Only for special cases can the sampled network have a similar structure to the complete network [49], while the solution that excludes node 8 can be the special case that is also the best solution of D*k*SP. If we exclude node 8, most of edges can be preserved because of the principle of largest density.

To solve D*k*SP, a number of studies have focused on the use of semidefinite programming; that is, the problem is transformed into a semidefinite programming problem for each node of a branch-and-bound tree [39, 40]. Some semidefinite programming relaxations have been also used to approximate D*k*SP [50, 51]. Other studies wrote D*k*SP as a problem of rank-constrained cardinality minimization, and relaxed it by the use of the nuclear norm [52, 53]. Also, a series of heuristic algorithms have been employed in solving the problem. Kincaid proposed a simulated annealing algorithm and a tabu search algorithm to solve the NP-hard D*k*SP [54]. Macambira employed a tabu search algorithm which was shown to outperform greedy search [55]. A variable neighborhood search heuristic proposed by Brimberg et al. was shown to be effective in solving the D*k*SP [56].

From a sociological view, given that inappropriate boundary specification can have a detrimental effect on estimating the structure of a real population, a list of sampling methods related to the sampling in network surveys has been also proposed. For example, randomly selecting individuals is a common method of sampling in social science investigations [27, 45, 57]. Top-down sampling (choosing the top nodes ordered by size) has also been widely used and yields estimates of network properties that are highly consistent with those obtained from whole network analysis [58, 59].

### 2.2. Covering problem with multiple graphs

Finding multiple densest subgraphs has recently been discussed [23–25, 60]. Balalau et al. focused on finding a set of *m* subgraphs that maximize the total density of each subgraph with the constraint of an upper bound on the pairwise Jaccard coefficient between the sets of nodes of the subgraphs (denoted as “multiple-*m* densest subgraphs problem”, M*m*DSP) [23]. Nasir et al. proposed a dynamic variant of this problem, where a collection of *m* disjoint subgraphs is found in a sliding window [25]. An approach similar to M*m*DSP was proposed by Galbrun et al. where the objective function takes both the total density and the distance between the subgraphs into account [24]. Dondi et al. addressed the approximability and computational complexity of this problem [60]. An application of M*m*DSP on dual networks has been also studied [61]. In this paper, we study the multiple-*m* densest subgraphs problem (M*m*DSP) proposed by Balalau et al. [23]. M*m*DSP aims to find a collection of *m* subgraphs {*G*^*i*^(*V*^*i*^,*E*^*i*^)} for which the sum of the average density of each subgraph $\frac{\left|E^{i}\right|}{\left|V^{i}\right|}$ is maximized [23]. Optimization of M*m*DSP can be formulated as problem (3) below, where *a* is the upper bound on the pairwise Jaccard coefficient.


$$\text{Maximize}\;\sum_{i}^{m}\frac{\left|E^{i}\right|}{\left|V^{i}\right|},\;\text{s}.\text{t}.\frac{\left|V^{i}\cap V^{j}\right|}{\left|V^{i}\cup V^{j}\right|}\leq a,\;\forall V^{i},V^{j}\subseteq V$$ (3)

M*m*DSP has focused on improving the density of each subgraph but has ignored the covering of the input network by extracting subgraphs. Although techniques such as the pairwise Jaccard coefficient or the distance between subgraphs have been invoked to avoid too much overlap between the extracted subgraphs [23, 24], the literature still lacks a focus on the network covering problem. The present study aims to find an optimal method for finding multiple subgraphs that best cover the input network, denoted as M*m*C*k*SP. There are three key elements associated with the sampling process: the covering of the input network (*C*), the bound on the subgraph size (*k*), and the number of subgraphs (*m*). Given the size of each subgraph *|V*^*i*^*|*, practitioners need to assemble the collected ties into a network that can best cover the input network (*C*). The objective function of M*m*C*k*SP is then formulated as (4), below. When the limited number of subgraphs is 1, problem (4) can be transformed to problem (2).


$$\text{Maximize}\;C(E_{1},E_{2},\ldots ,E_{m}),\;\text{s}.\text{t}.\forall \left|V^{i}\right|\leq k,\;\forall i\leq m$$ (4)

**Fig 4:**
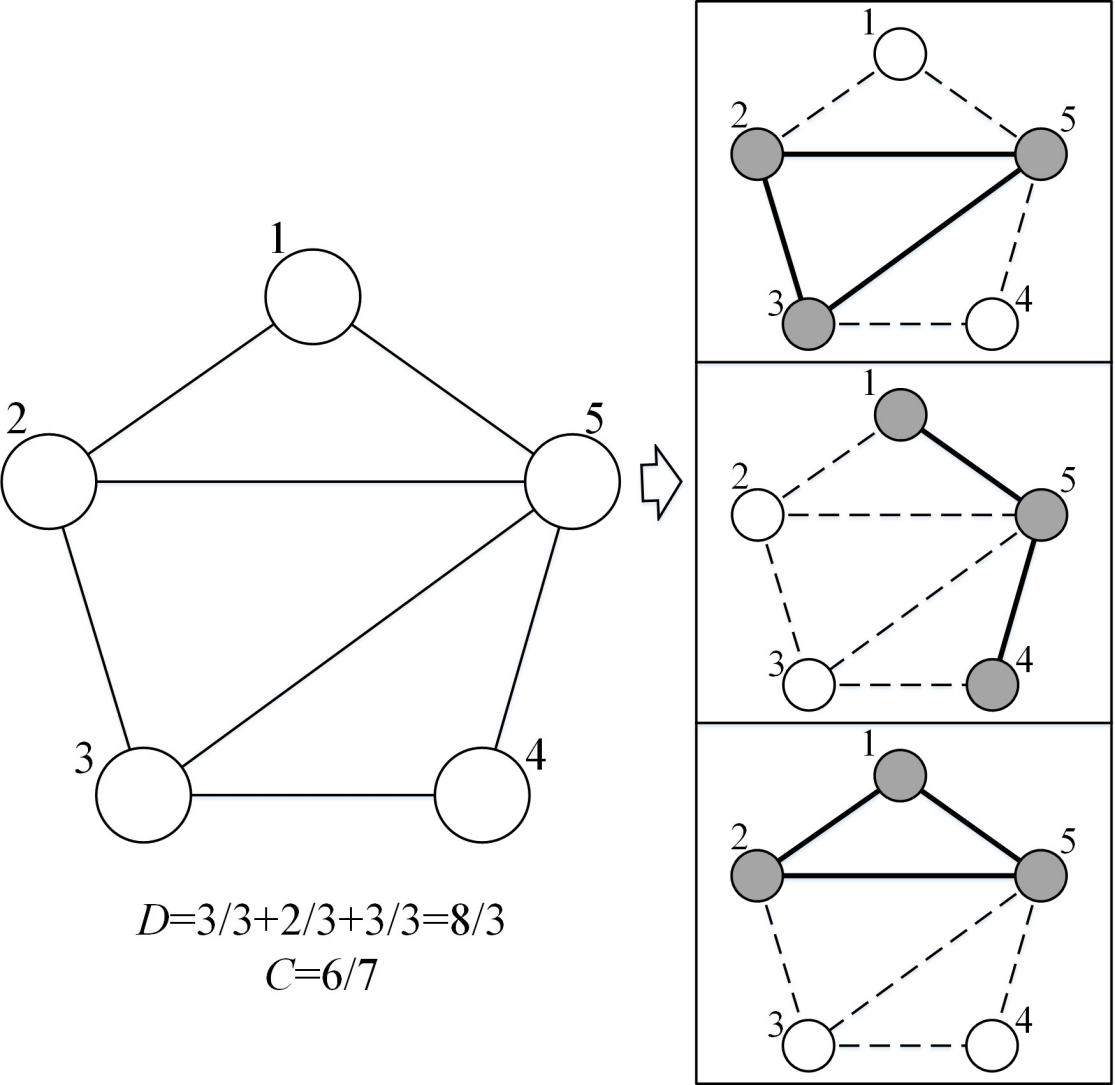
Computing the objective function. The left part is the input network which consists of five nodes and seven edges. The right part is a solution containing three subgraphs, where grey nodes represent the included nodes and white nodes represent the omitted nodes. Dashed lines represent unobserved ties, while bold solid lines represent the extracted ties. The sum of the average density of this solution is 8/3, while the covering of the input network is 6/7, because only six edges (excluding edges between nodes 3 and 4) have been extracted.

Here we use the fraction of extracted edges to measure *C*, i.e., C(*E_1_*,*E*_2_,…,*E*_*m*_) = cover(…cover(cover(*E*_1_,*E*_2_),*E*_3_),…,*E*_*m*_)/|*E*|, where cover(*E*_*i*_, *E*_*j*_) = |*E*_*i*_ ∪ *E*_*j*_| − |*E*_*i*_ ∩ *E*_*j*_|. An illustration of the measurement of *C* is shown in Fig 4. Compared with problem (3), *C* contains the physical significance of *a* (a parameter for avoiding subgraphs being too similar in problem (3)). Since the objective is to maximize the total covering of the input network, the extracted subgraphs should be different, and thus we do not necessarily employ *a* in problem (4).

**Fig 5:**
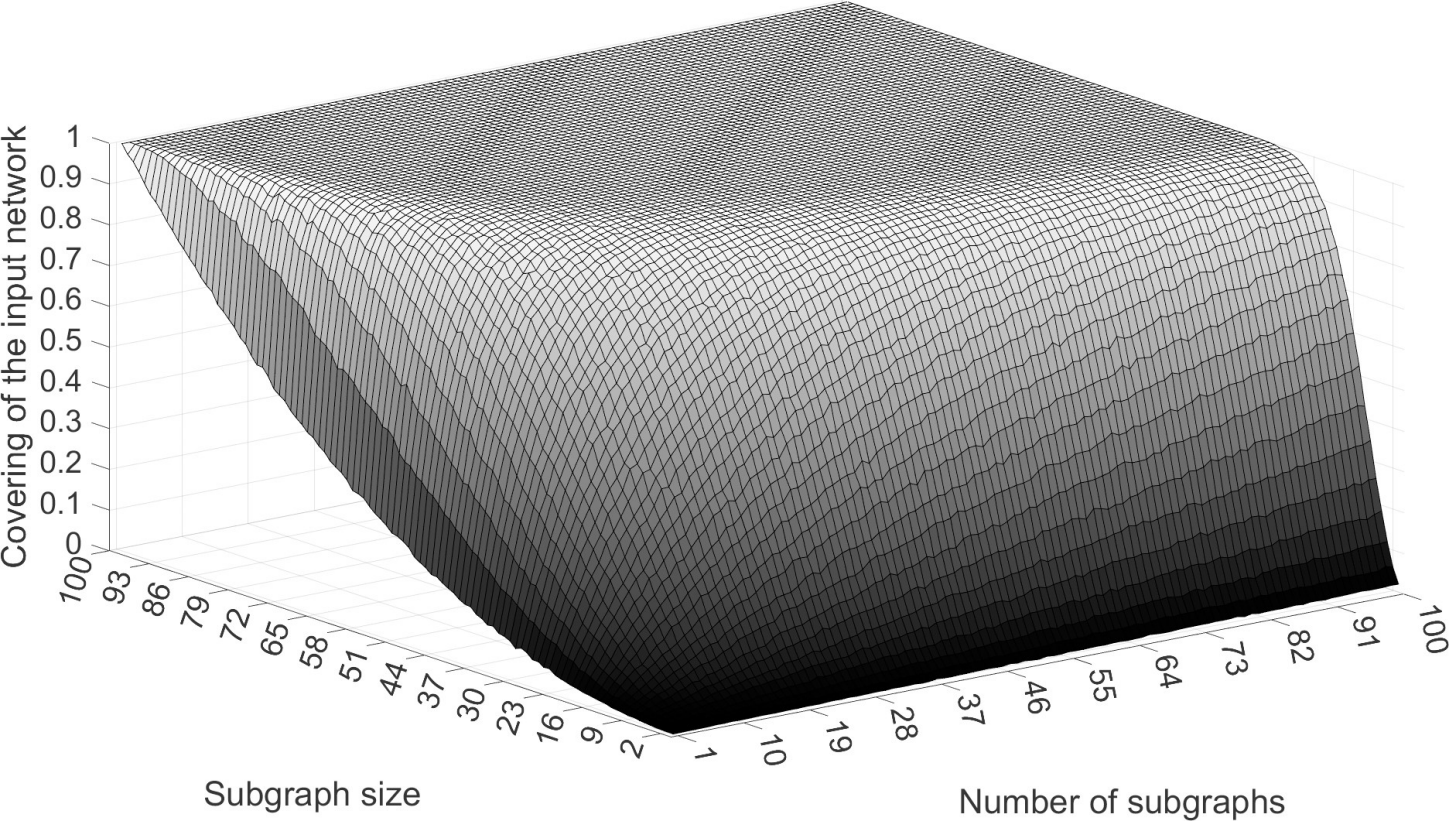
Relationship between the three key elements in the sampling process. X-axis, Y-axis and Z-axis are, respectively, the subgraph size, the number of subgraphs and the covering of the input data. The simulation was performed on an ER random network with 100 nodes and 1,000 edges. The mean value of the results across 10 runs is presented in the figure.

The functional relationship between the three elements listed above is non-linear as can be seen from a simulation of the random sampling shown in Fig 5. We find that increasing subgraph size is more helpful in promoting representativeness than increasing the number of subgraphs, because the gradient $\frac{dC}{dk}$ is greater than $\frac{dC}{dm}$.

## 3. Algorithm

Given a fixed number of subgraphs (*m*), subgraph size (*k*) and the entire population (*N*), the number of possible extracted subgraphs is

$$\left(\begin{array}{c} \left(\begin{array}{c} N\\ k \end{array}\right)\\ m \end{array}\right)=\frac{(\frac{N!}{k!(N-k)!})!}{m![(\frac{N!}{k!(N-k)!})!-m]!}.$$

Traversing all these solutions cannot be computed polynomial time, and thus M*m*C*k*SP constitutes an NP-hard problem. Compared with the NP-hard M*m*DSP proposed by Balalau et al. [23], M*m*C*k*SP is more complicated because of the higher time cost for computing the covering in place of the average density, as well as setting the bound *k* on subgraph size. In this section, we introduce a memetic algorithm that combines a genetic algorithm and a heuristic local search called the memetic algorithm to find multiple subgraphs that cover the input network (MA-M*m*C*k*SP). The memetic operation includes both long-distance and short-distance search and has proved to be effective in solving NP-hard problems [62, 63].

### 3.1. Framework

The framework of MA-M*m*C*k*SP is shown in Algorithm 1. We first input necessary parameters and the adjacency matrix of the input network. An initial population *P* is generated that consists of a list of solutions (coded as chromosomes), and then the process is repeated until the maximum number of iterations is reached or the coverage of the input network remains unchanged over 50 iterations. At each iteration, tournament selection is used to select a parent population *P*_*parent*_ with the highest representativeness. Next, we perform a genetic operation on *P*_*parent*_ to form an offspring population *P*_*offspring*_. Then the local-search function is applied to find the local maximum solution for the offspring population. Then an updating function is used to construct a new population *P* with better solutions. After repeating, we output the fittest solution by decoding.

#### Algorithm 1. Framework of MA-M*m*C*k*SP

1. Input: Population size (*S*_*p*_), Tournament size (*S*_*tour*_), Mating pool size (*S*_*pool*_), Crossover probability (*P*_*c*_), Maximum number of iterations (*M*_*i*_), Number of nodes (*N*), Number of subgraphs (*m*), subgraph size (*k*), Adjacency matrix of networks (*A*).
2. *P* ← Initialization (*S*_p_, *N*, *m*, *k*);
3. Repeat
4.  *P*_*parent*_ ← Selection (*P*, *S*_*tour*_, *S*_*pool*_);
5.  *P*_*offspring*_ ← Genetic Operation (*P*_*parent*_, *P*_*c*_, *P*_*m*_, *N*, *m*, *k*);
6.  *P*_*offspring*_ ← Local Search (*P*_*offspring*_, *N*, *m*, *k*);
7.  *P* ← Update (*P*, *P*_*parent*_, *P*_*offspring*_);
8. Until Termination (*I*_*max*_)
9. Decode (*P*)
10. Output: the best solution of the finding multiple subgraphs and its covering.

### 3.2. Representation and initialization

**Fig 6:**
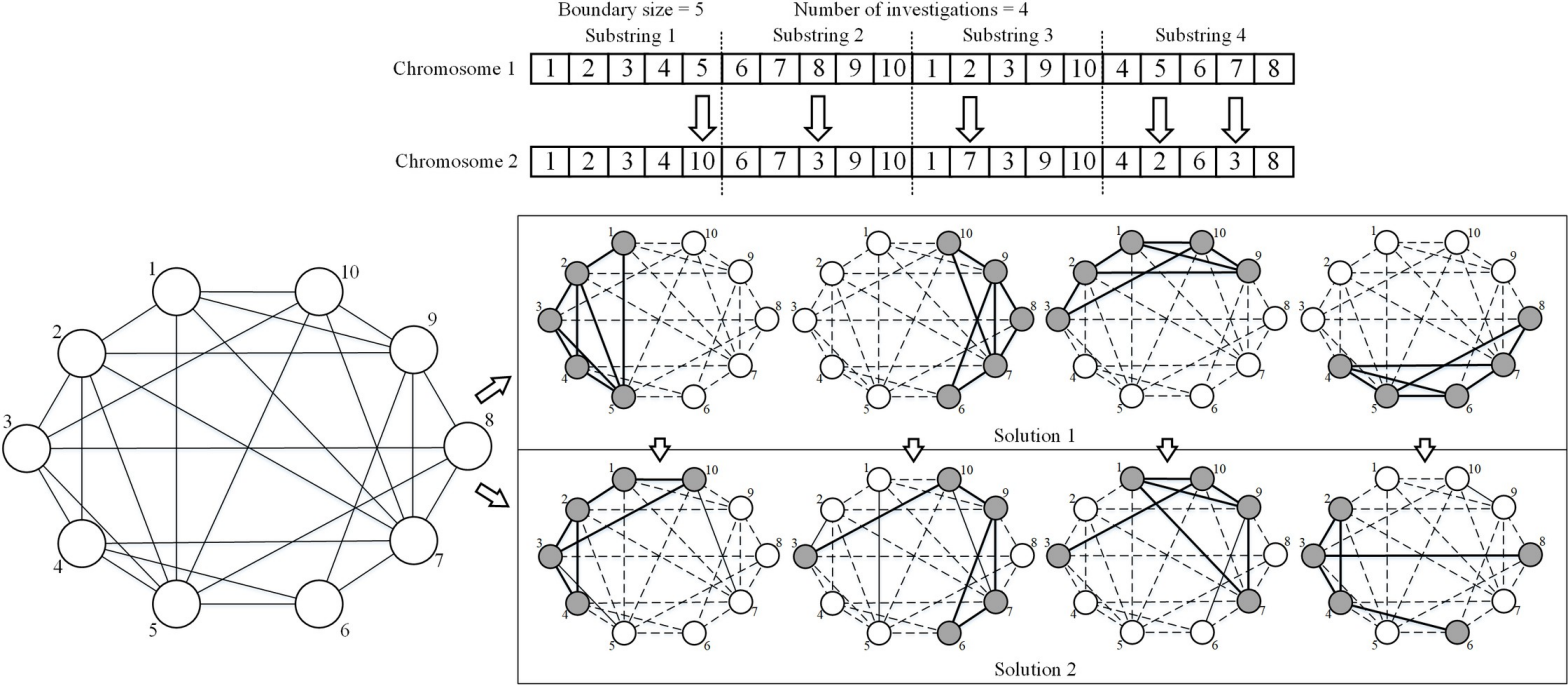
Illustration of the representation. The upper two chains denote two chromosomes consisting of genes. The lower left part is an input network, which consists of ten nodes. The lower right part is two solutions of extracted multiple subgraphs corresponding to the two chromosomes, where grey nodes represent the included nodes and white nodes represent the omitted nodes. The dashed line represents unobserved ties, while the bold solid lines represent the extracted ties.

Each solution is encoded as a chromosome that consists of *m* substrings *X* = [*X*_1_, *X*_2_, …, *X*_*m*_], where *m* is the number of subgraphs. Each substring represents the node set in a subgraph and is denoted by a list of genes *x* ∈ {1, 2, …, *n*} that specifies which nodes should be included. Fig 6 illustrates the representation for a subgraph of size 5, and the number of subgraphs is set to 4, so the chromosome is formed as five genes with four substrings. If we change the 5^th^ gene from 5 to 10 in the first substring, the new solution will substitute node 10 for node 5 in the first subgraph.

For the initialization, we generate a population and randomly select the nodes for each substring in every chromosome.

### 3.3. Genetic operation

The genetic operation includes both crossover and mutation, which are the primary operations in the genetic algorithm. The algorithm performs the crossover procedure with probability *P*_*c*_, and executes the mutation procedure with probability *P*_*m*_ = 1−*P*_*c*_. To some extent crossover represents long-term search, while mutation represents short-term search. Thus appropriate setting of *P*_*m*_ = 1−*P*_*c*_ enables a balance to be found between long-term and short-term search, which helps to increase the efficiency of the genetic algorithm [64, 65].

**Fig 7:**
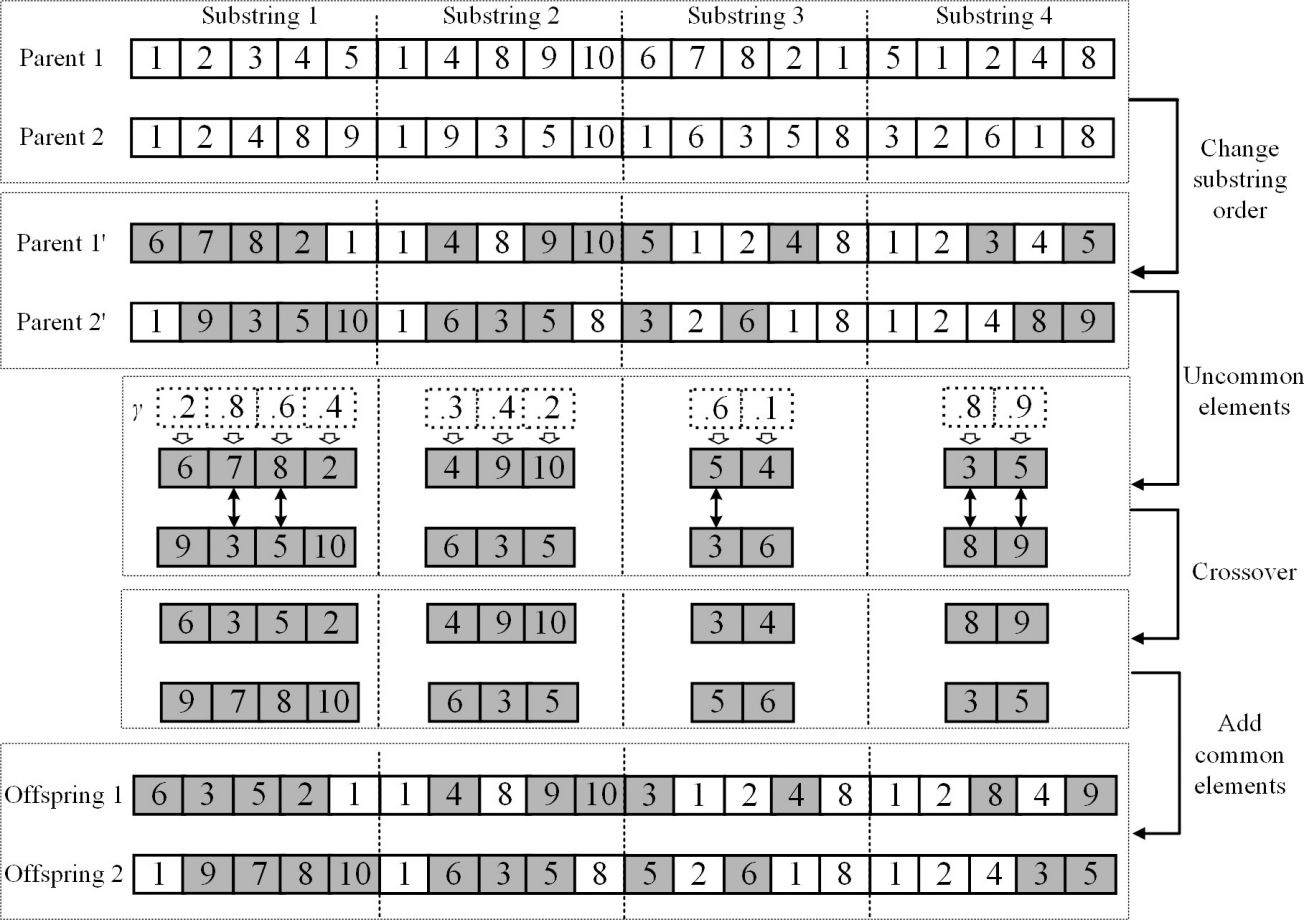
The crossover operation. Grey elements represent genes that differ between the two parental chromosomes. The substrings of two parent chromosomes are first disorganized, and we check all elements that differ between the two parental chromosomes. If the random number *γ* < 0.5, the element remains unchanged in the offspring chromosomes, while if *γ* ≥ 0.5, the corresponding elements are swapped.

In the crossover operation, two parental chromosomes are chosen using tournament selection. We first disorganize the order of the substrings for each chromosome to maintain diversity, and then find the genes that differ between the chromosomes in each substring. Given each pair of different genes, we generate a random number *γ*; if *γ*< 0.5, the gene remains unchanged; and if *γ* ≥ 0.5, the corresponding genes are swapped between the two chromosomes. Finally, we add the common genes and form the two offspring chromosomes. The crossover operation is illustrated in Fig 7. After changing the substring disorder, substring 3 in parent 1 and substring 2 in parent 2 are reassigned to the first substring. The genes that differ between parent 1*’* and parent 2*’* are grey. Since the generated random numbers are 0.3, 0.6, 0.9, and 0.4, respectively, for the first substring, we swap the second and third different genes between the two parental chromosomes because the corresponding *γ* ≥ 0.5.

In the mutation operation, we randomly select an element *x*_*i*_ in each substring and then randomly assign a different node number that is also different from other node numbers within the same substring as the element *x*_*i*_.

### 3.4. Local search

Local search is effective in reducing inefficient exploration and not only improves the accuracy but also speeds up the convergence [64–66]. Here we employ a hill-climbing technique presented as Algorithm 2. We check each element in a chromosome and replace the original gene with a node number that increases the objective function (the coverage of the input network) on substitution. The chromosome can then reach a local optimum.

#### Algorithm 2. Local Search in MA-M*m*C*k*SP

1. Input: The best offspring chromosome (*C*_*offspring*_), number of nodes (*N*), number of subgraphs (*m*) and subgraph size (*k*);
2. For *i* = 1; *i* ≤ *k* × *m*; *i*++
3.  *C*_*offspringnew*_ = *C*_*offspring*_
4.  For *j* = 1; *j* ≤ *N*; *j*++
5.   *C*_*offspringnew*_(*i*) = *j*;
6.   If Obj(*C*_*offspringnew*_) > Obj(*C*_*offspring*_)
7.    *C*_*offspring*_(*i*) = *j*;
8.   End If
9.  End For
10. End For
11. Output: *C*_*offspring*_

### 3.5. Complexity analysis

Given a network with *N* nodes, number of subgraphs *m* and the subgraph size *k*, the time-complexity of MA-M*m*C*k*SP is analyzed as follow. At each iteration, we need to execute the crossover operation S_*pool*_/2 times (where S_*pool*_ is the size of the mating pool) and the mutation operation S_*pool*_ times at most. Since computing the covering costs *O*(*mk*), the time-complexity for performing the genetic operation is *O*(*mkS*_*pool*_). In the local search procedure, finding the best neighbor for each gene needs *O*(*Nmk*), and thus to find the local optimal chromosome will cost *O*(*Nm*^2^*k*^2^). Since *O*(*mkS*_*pool*_) < *O*(*Nm*^2^*k*^2^), the total time complexity of the proposed algorithm is *O*(*Nm*^2^*k*^2^).

## 4. Results

In this section, we show the effectiveness and efficiency of MA-M*m*C*k*SP running on a computer-generated random network. We also carry out the procedure on various real-world networks and interpret the optimal method in the social context. The experiments were carried out on a 2.11 GHz CPU with 16.00 GB memory computer, running on Windows 10 using MATLAB to execute the procedure. Table 1 shows the parameters used in the experiments that gave the best performance for the proposed algorithms.

**Table 1:** Parameters in the experiments.

Parameter	Meaning	Value
*M*_*i*_	Maximum Iterations	1000
*S*_*p*_	Size of population	200
S_*pool*_	Mating pool size	100
*S*_*tour*_	Size of tournament	2
*P*_c_	Probability of Crossover	0.8

### 4.1. Results for computer-generated networks

**Fig 8:**
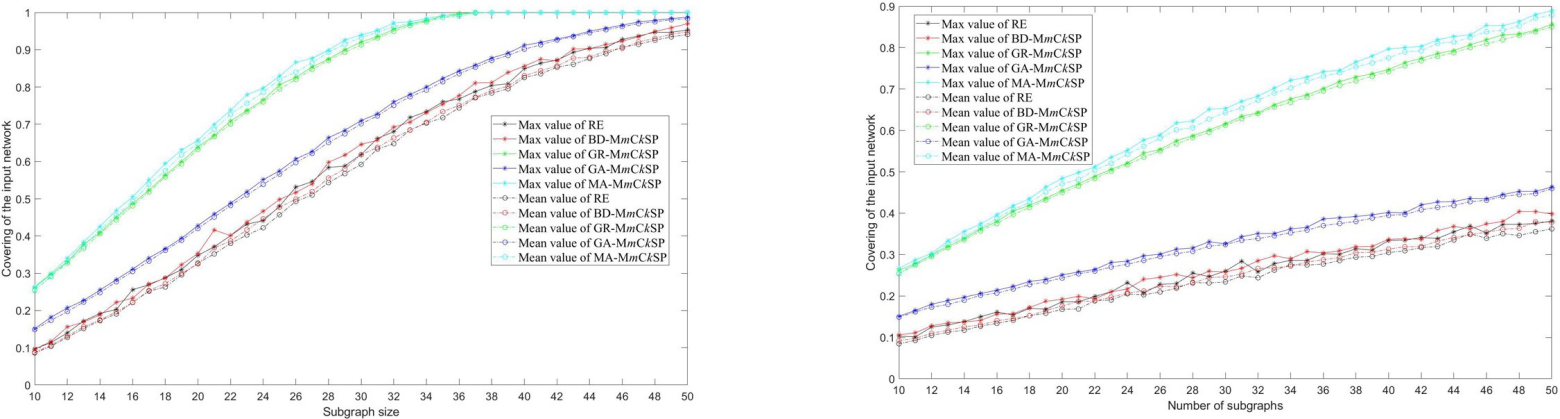
Covering the input random network with RE, BD-M*m*C*k*SP, GR-M*m*C*k*SP, GA-M*m*C*k*SP and MA-M*m*C*k*SP. (a) shows the result for different subgraph sizes given the number of subgraphs *m* = 10; (b) shows the result for different numbers of subgraphs given the subgraph size *k* = 10. The solid line with stars represents the maximum value of the ten-runs experiment, while the dashed-dotted line with the circles represents the mean value.

In order to assess the effectiveness of MA-M*m*C*k*SP, we compare it with random extraction (RE), the big-degree sampling method where big-degree nodes have a higher probability of being extracted (BD-M*m*C*k*SP), the greedy algorithm based on the operation of local search (GR-M*m*C*k*SP) and the genetic algorithm without local search (GA-M*m*C*k*SP). The five methods were carried out on an ER random network consisting of 100 nodes and 1,000 edges. Fig 8A and 8B present the maximum and mean values of 10-runs, comparing covering of the input network for different settings of subgraph size and number of subgraphs. The figures show that MA-M*m*C*k*SP performs the best, GR-M*m*C*k*SP performs second best, GA-M*m*C*k*SP performs the third, while BD-M*m*C*k*SP and RE perform the worst. From Fig 8A we see that the whole network’s ties can be collected using only 10 subgraphs when the subgraph size reaches 37, which is much smaller than the theoretical maximum $\left(\begin{array}{c} 100\\ 37 \end{array}\right)$. We conclude that MA-M*m*C*k*SP is effective in solving the optimization problem of sampling in multiple social surveys. The slopes of the curves in Fig 8A are much higher than those in Fig 8B, which suggests that focusing on subgraph size is more important than focusing on numbers of subgraphs.

**Fig 9:**
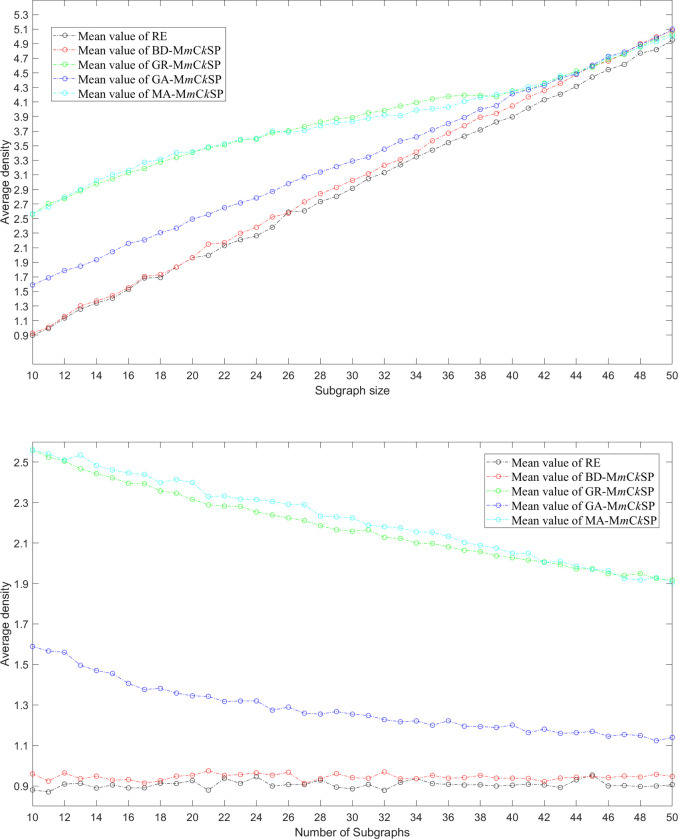
Average density of the extracted subgraphs for RE, BD-M*m*C*k*SP, GR-M*m*C*k*SP, GA-M*m*C*k*SP and MA-M*m*C*k*SP. (a) shows the result for different subgraph sizes given the number of subgraphs *m* = 10; (b) shows the result for different numbers of subgraphs given the subgraph size *k* = 10.

We computed the average density of the extracted solutions corresponding to Fig 8. Fig 9A and 9B present the mean values of the average density in 10-runs for different settings of subgraph size and number of subgraphs. The figures show that the extracted subgraphs derived from MA-M*m*C*k*SP are densest, GR-M*m*C*k*SP are the second densest, GA- M*m*C*k*SP are the third densest, while subgraphs in BD-M*m*C*k*SP and RE are sparsest. The results suggest that the optimal extracted subgraphs are more likely to be denser. The proposed algorithm can provide a new alternative for solving the multiple-*m* densest subgraphs problem (M*m*DSP). In addition, we find the density of subgraphs increases as the subgraph size increases, but decreases as the number of subgraphs decreases. There is a tradeoff among the subgraph density, subgraph size and the number of subgraphs.

**Fig 10:**
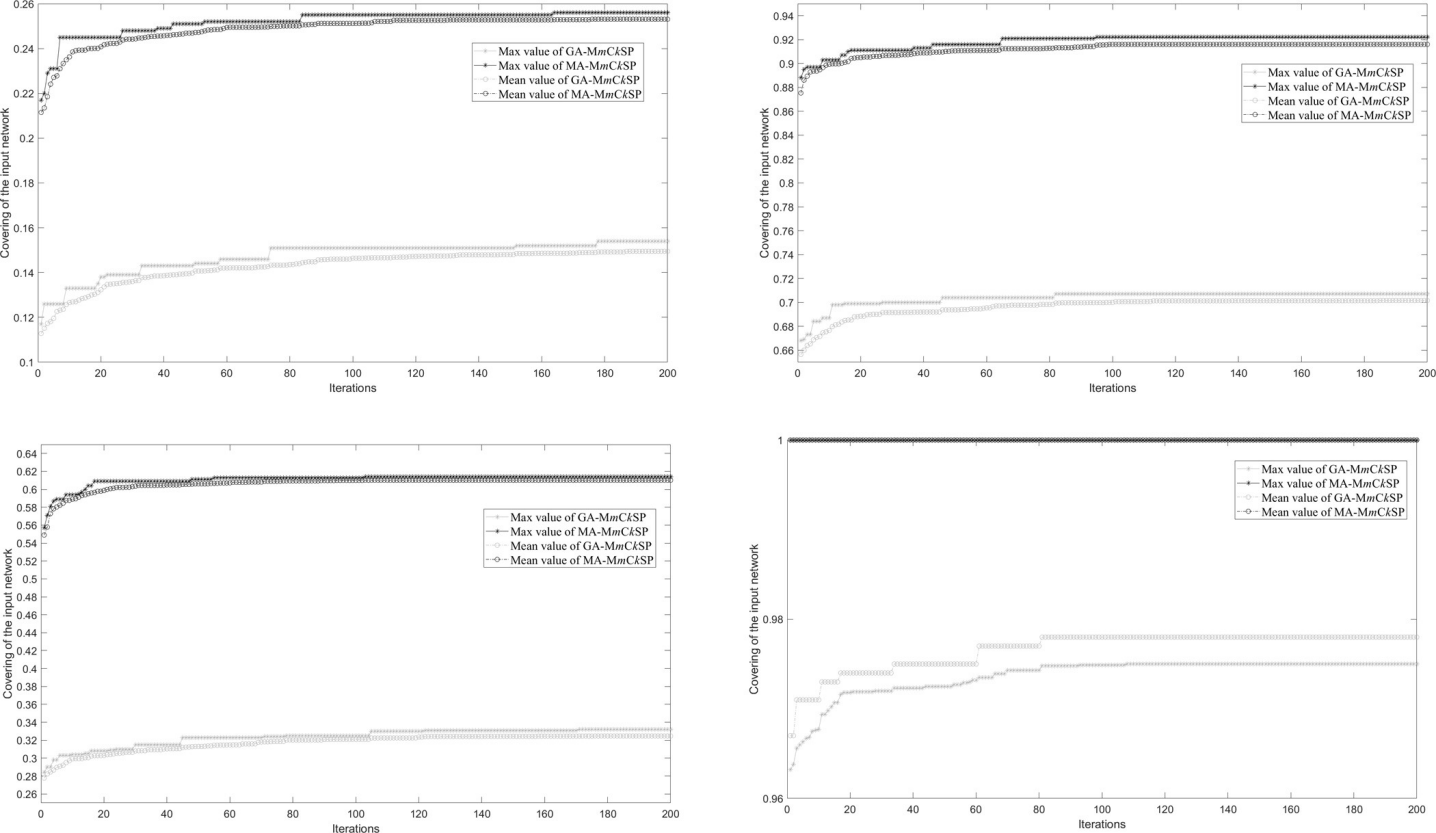
Covering of the input random network with GA-M*m*C*k*SP and MA-M*m*C*k*SP for each iteration. Figures (a)-(d) are, respectively, the results for the settings *k* = 10 and *m* = 10, *k* = 10 and *m* = 30, *k* = 30 and *m* = 10, *k* = 30 and *m* = 30. The solid line with the stars represents the maximum value of the ten-runs experiment, while the dashed-dotted line with the circles represents the mean value. Grey and black, respectively, represent the results of GA-M*m*C*k*SP and MA-M*m*C*k*SP.

We also compared the results obtained using MA-M*m*C*k*SP with those using GA-M*m*C*k*SP on each iteration, and we see that the memetic operation is more efficient. Fig 10 show the results for the two methods with different settings for subgraph size and number. MA-M*m*C*k*SP performs much better and converges faster than GA-M*m*C*k*SP. The difference is especially apparent in Fig 10D, where MA-M*m*C*k*SP is able to reach a covering of 100% at the first iteration, while GA-M*m*C*k*SP converges after the 80^th^ iteration and even then does not reach 100%.

**Fig 11:**
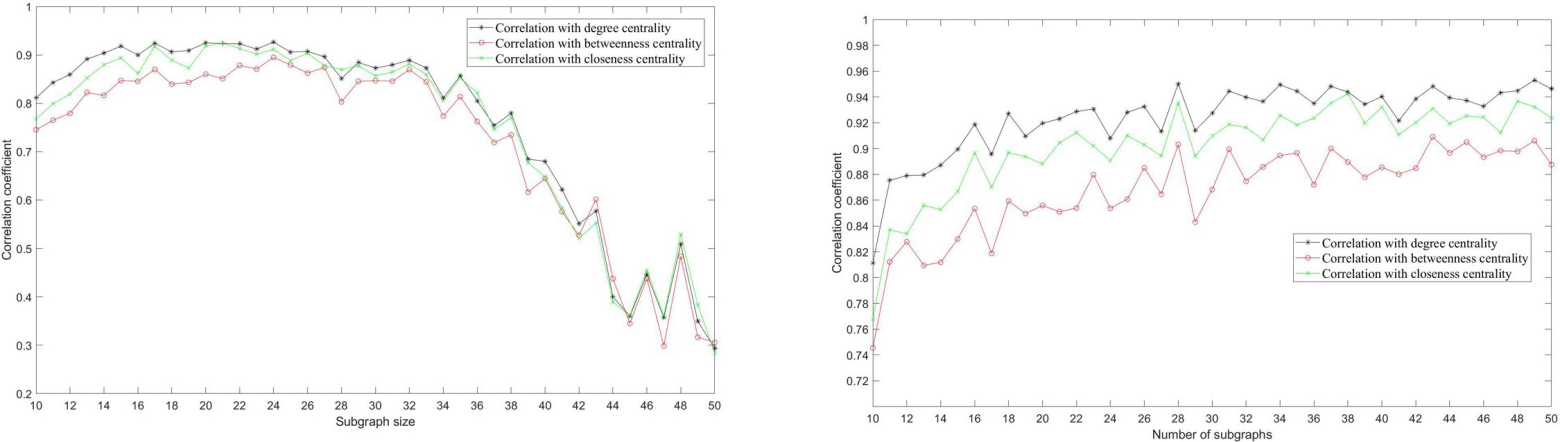
Correlation between the number of times each node is selected and the network centrality corresponding to Fig 6. (a) shows the result for different subgraph sizes given the number of subgraphs *m* = 10; (b) shows the result for different number of subgraphs given the subgraph size *k* = 10. The different curves represent the correlations with degree centrality, betweenness centrality and closeness centrality.

In order to find characteristics of the extracted nodes, we compute the correlation between the number of times each node is selected using MA-M*m*C*k*SP and the network centrality, as shown in Fig 11. Comparing Figs 8A and 11A, when the network cannot be completely collected (i.e., the subgraph size is smaller than 37), the probability of a node being selected is highly correlated with its centrality. The correlation dramatically decreases as the boundary size surpasses the critical value. Fig 11B shows a similar result if central nodes are more likely to be included repeatedly. The results suggest that including central nodes is helpful in achieving the network covering.

**Fig 12:**
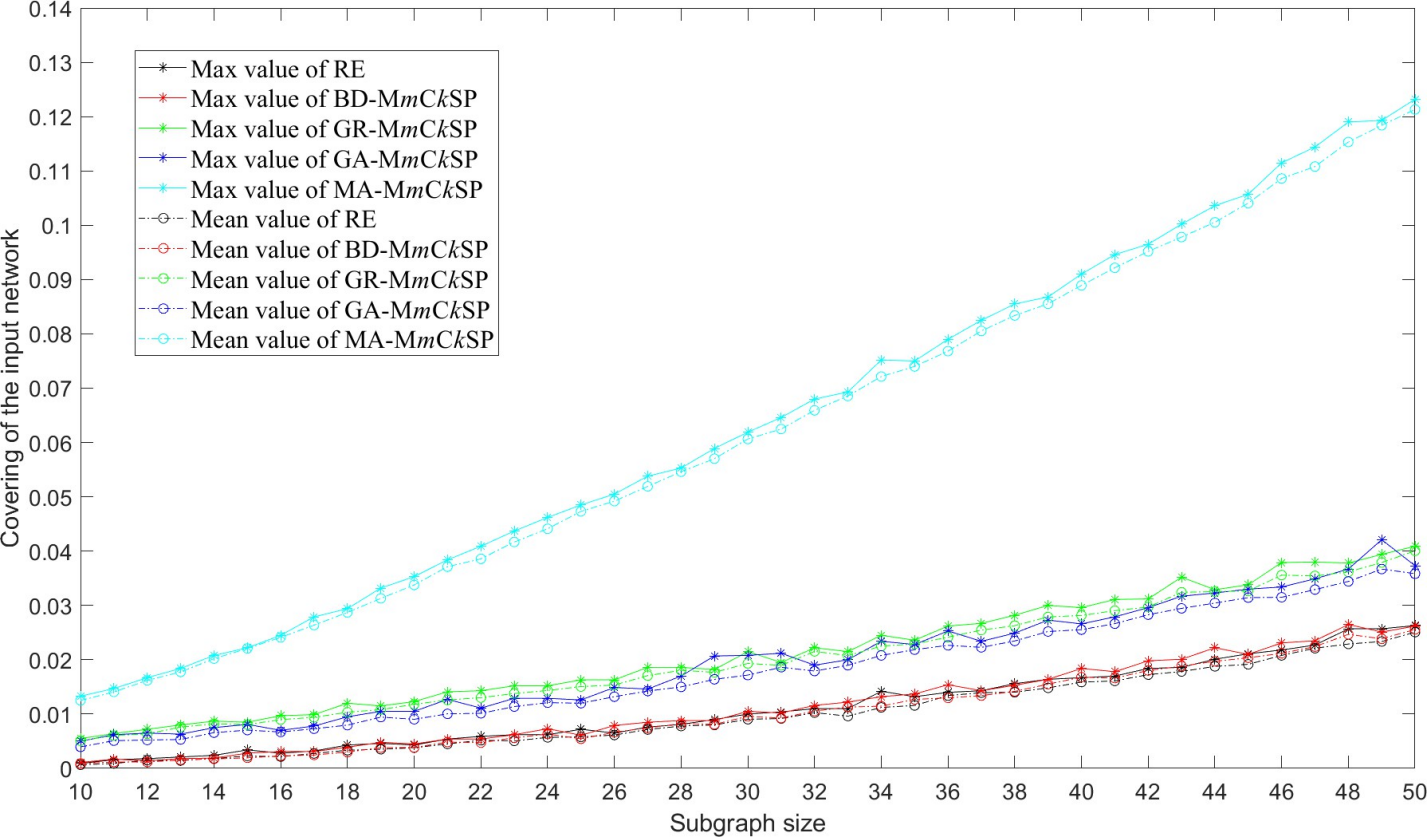
Covering of the 1000-node input random network acquired by RE, BD-M*m*C*k*SP, GR-M*m*C*k*SP, GA-M*m*C*k*SP and MA-M*m*C*k*SP for different subgraph sizes given the number of subgraphs *m* = 10.

**Fig 13:**
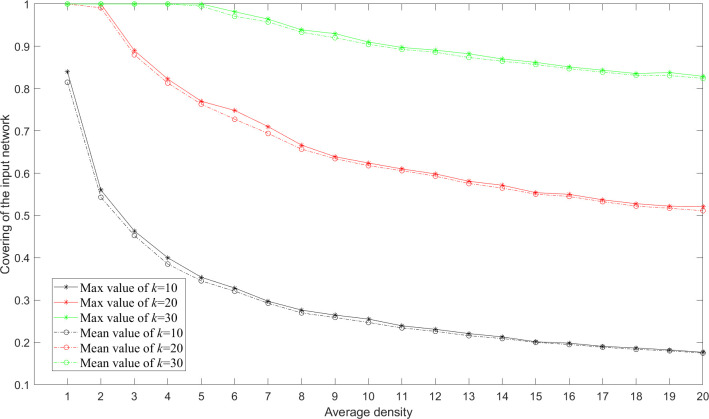
Covering of the input random 100-node network with different average densities given the number of subgraphs *m* = 10 and the subgraph size *k* = 10, 20 and 30 acquired by MA-M*m*C*k*SP.

A sensitivity analysis for the proposed algorithm on a network with 1,000 nodes and 10,000 edges is conducted. The results show that MA-M*m*C*k*SP still performs the best in maximizing the coverage of networks of larger size as shown in Fig 12. We also test the performance of MA-M*m*C*k*SP for networks with different average densities and find that the extracted subgraphs are less covering given the fixed number of subgraphs and the subgraph size when the density of the input network increases as shown in Fig 13. A subgraph of larger size is required if we aim to investigate a denser social network.

### 4.2. Results for real-world networks

In this section, we test RE, GA-M*m*C*k*SP and MA-M*m*C*k*SP on six real-world networks, namely Zachary’s Karate Club network, Bottlenose Dolphins network, American College Football network and three migrant workers’ networks of ADS, YDSC and WH companies in Shenzhen, China.

Zachary’s Karate Club network consists of 34 karate-club members and 78 social ties observed by Zachary over two years [67]. The Bottlenose Dolphins network was constructed by Lusseau [68], who observed 62 bottlenose dolphins and their 159 connections over seven years. The American College Football network was constructed from the schedule of Division Ⅰ games during the year 2000 football season. The network consists of 115 nodes that represent teams and 616 edges that represent the regular season games between the two teams that they connect [7]. The next three examples are networks of migrant workers in ADS, YDSC, and WH companies investigated by the New Urbanization and Sustainable Development Group of Xi’an Jiaotong University [65]. The three networks were constructed from a single-item question that asked the participant to enumerate individuals with whom they are often in contact at work. ADS network consists of 165 nodes and 1196 edges; YDSC network consists of 70 nodes and 272 edges; WH network consists of 193 nodes and 887 edges. The survey involved both network-level and individual-level investigations.

For each network, the number of subgraphs is *m* = 5, 10, or 30, and the subgraph size is chosen from *k* = 0.1*N*, *k* = 0.2*N*, *k* = 0.3*N*, *k* = 0.4*N*, *k* = 0.5*N*, where *N* is the network size. Table 2 shows the mean and maximum value of representativeness over 10 runs produced by RE, BD-M*m*C*k*SP, GR-M*m*C*k*SP, GA-M*m*C*k*SP and MA-M*m*C*k*SP with different values of *m* and *k*. We find MA-M*m*C*k*SP performs much better than other algorithms. Moreover, subgraph size *k* plays a much more important role in the multiple extractions: a small increase in *k* can produce a large improvement in covering. Even random extraction is able to cover all the edges when *k* reaches 0.4*N*.

**Table 2:** Mean and maximum values of the covering for the real-world networks.

Networks	Index	*k =* 0.1*N*			*k =* 0.2 *N*			*k =* 0.3 *N*			*k =* 0.4 *N*			*k =* 0.5 *N*		
		*m* = 5	*m* = 10	*m* = 30	*m* = 5	*m* = 10	*m* = 30	*m* = 5	*m* = 10	*m* = 30	*m* = 5	*m* = 10	*m* = 30	*m* = 5	*m* = 10	*m* = 30
Karate	RE-max	0.051	0.115	0.180	0.231	0.423	0.769	0.423	0.667	0.949	0.718	0.936	1	0.872	0.949	1
	BD-max	0.051	0.115	0.256	0.295	0.526	0.846	0.577	0.795	1	0.795	0.936	1	0.885	1	1
	GR-max	0.192	0.385	0.897	0.756	1	1	0.987	1	1	1	1	1	1	1	1
	GA-max	0.192	0.359	0.590	0.718	0.833	1	0.910	0.974	1	1	1	1	1	1	1
	MA-max	0.192	0.385	0.936	0.769	1	1	1	1	1	1	1	1	1	1	1
	RE-mean	0.028	0.050	0.110	0.162	0.314	0.686	0.322	0.558	0.896	0.626	0.865	0.994	0.777	0.912	1
	BD-mean	0.035	0.077	0.186	0.241	0.432	0.781	0.444	0.673	0.968	0.727	0.887	0.997	0.823	0.982	1
	GR-mean	0.185	0.362	0.840	0.723	0.973	1	0.937	1	1	1	1	1	1	1	1
	GA-mean	0.192	0.315	0.545	0.644	0.776	0.971	0.839	0.942	1	0.973	1	1	1	1	1
	MA-mean	0.192	0.385	0.924	0.768	1	1	0.999	1	1	1	1	1	1	1	1
Dolphins	RE-max	0.076	0.113	0.270	0.220	0.384	0.761	0.459	0.660	0.969	0.679	0.855	1	0.774	0.962	1
	BD-max	0.075	0.119	0.346	0.252	0.384	0.774	0.522	0.767	0.987	0.736	0.906	1	0.918	0.969	1
	GR-max	0.358	0.604	1	0.761	0.969	1	0.962	1.000	1	1	1	1	1	1	1
	GA-max	0.315	0.390	0.541	0.654	0.667	0.899	0.881	0.899	1	0.912	0.981	1	0.969	1	1
	MA-max	0.377	0.610	1	0.793	1	1	1	1	1	1	1	1	1	1	1
	RE-mean	0.040	0.084	0.203	0.157	0.294	0.664	0.379	0.587	0.945	0.603	0.830	0.996	0.747	0.935	0.999
	BD-mean	0.057	0.095	0.274	0.216	0.354	0.738	0.469	0.692	0.964	0.658	0.881	0.994	0.853	0.959	0.999
	GR-mean	0.325	0.569	0.978	0.725	0.932	1	0.933	1	1	0.998	1	1	1	1	1
	GA-mean	0.297	0.369	0.518	0.616	0.653	0.894	0.806	0.882	1	0.893	0.979	1	0.964	1	1
	MA-mean	0.367	0.598	1	0.781	0.994	1	0.996	1	1	1	1	1	1	1	1
Football	RE-max	0.070	0.131	0.286	0.201	0.343	0.726	0.398	0.626	0.963	0.600	0.842	0.997	0.781	0.959	1
	BD-max	0.055	0.113	0.289	0.207	0.359	0.708	0.401	0.641	0.951	0.592	0.848	1	0.791	0.961	1
	GR-max	0.378	0.701	0.948	0.765	0.946	1	0.943	1	1	0.995	1	1	1	1	1
	GA-max	0.264	0.212	0.379	0.463	0.447	0.808	0.692	0.736	0.989	0.729	0.914	1	0.883	0.992	1
	MA-max	0.370	0.635	0.966	0.768	0.941	1	0.966	1	1	1	1	1	1	1	1
	RE-mean	0.053	0.098	0.261	0.184	0.321	0.692	0.371	0.613	0.941	0.569	0.824	0.995	0.761	0.942	1
	BD-mean	0.049	0.093	0.261	0.184	0.331	0.687	0.372	0.612	0.936	0.577	0.828	0.994	0.762	0.951	1
	GR-mean	0.349	0.667	0.930	0.710	0.927	1	0.925	1	1	0.980	1	1	1	1	1
	GA-mean	0.230	0.194	0.369	0.377	0.439	0.786	0.543	0.727	0.982	0.706	0.906	1	0.873	0.989	1
	MA-mean	0.357	0.615	0.956	0.747	0.932	1	0.952	1	1	1	1	1	1	1	1
ADS	RE-max	0.068	0.129	0.286	0.237	0.406	0.750	0.453	0.666	0.963	0.604	0.859	1	0.816	0.964	1
	BD-max	0.108	0.172	0.392	0.291	0.483	0.814	0.530	0.761	0.977	0.717	0.908	0.997	0.877	0.977	1
	GR-max	0.423	0.687	0.973	0.834	0.984	1	0.984	1	1	1	1	1	1	1	1
	GA-max	0.304	0.376	0.569	0.633	0.653	0.876	0.710	0.841	0.989	0.832	0.948	1	0.934	0.993	1
	MA-max	0.427	0.629	0.969	0.840	0.985	1	1	1	1	1	1	1	1	1	1
	RE-mean	0.049	0.094	0.259	0.185	0.338	0.687	0.370	0.625	0.935	0.564	0.817	0.996	0.747	0.945	1
	BD-mean	0.080	0.141	0.364	0.237	0.442	0.804	0.476	0.731	0.967	0.682	0.889	0.995	0.843	0.970	1
	GR-mean	0.402	0.663	0.959	0.789	0.973	1	0.968	1	1	0.996	1	1	1	1	1
	GA-mean	0.296	0.365	0.565	0.580	0.641	0.869	0.697	0.836	0.987	0.825	0.943	1	0.926	0.992	1
	MA-mean	0.421	0.616	0.962	0.827	0.975	1	0.996	1	1	1	1	1	1	1	1
YDSC	RE-max	0.088	0.132	0.279	0.217	0.379	0.710	0.434	0.636	0.952	0.651	0.875	1	0.842	0.985	1
	BD-max	0.096	0.154	0.364	0.268	0.485	0.827	0.515	0.801	0.971	0.728	0.912	1	0.919	0.974	1
	GR-max	0.324	0.574	0.974	0.765	0.960	1	0.967	1	1	1	1	1	1	1	1
	GA-max	0.309	0.393	0.596	0.625	0.699	0.915	0.820	0.886	1	0.901	0.978	1	0.982	1	1
	MA-max	0.338	0.574	0.993	0.787	0.993	1	0.993	1	1	1	1	1	1	1	1
	RE-mean	0.044	0.087	0.240	0.172	0.311	0.675	0.372	0.578	0.923	0.582	0.811	0.993	0.758	0.946	1
	BD-mean	0.074	0.122	0.314	0.235	0.416	0.782	0.456	0.725	0.956	0.678	0.876	0.994	0.835	0.958	1
	GR-mean	0.308	0.542	0.944	0.733	0.936	1	0.943	1	1	0.997	1	1	1	1	1
	GA-mean	0.284	0.368	0.559	0.566	0.668	0.897	0.747	0.867	0.997	0.872	0.969	1	0.957	1	1
	MA-mean	0.331	0.559	0.981	0.776	0.984	1	0.986	1	1	1	1	1	1	1	1
WH	RE-max	0.069	0.117	0.277	0.212	0.371	0.745	0.443	0.647	0.950	0.599	0.863	0.996	0.807	0.962	1
	BD-max	0.086	0.159	0.381	0.274	0.487	0.818	0.536	0.758	0.974	0.738	0.910	0.999	0.871	0.973	1
	GR-max	0.474	0.726	0.973	0.867	0.976	1	0.972	1	1	1	1	1	1	1	1
	GA-max	0.320	0.382	0.547	0.602	0.652	0.881	0.691	0.840	0.988	0.834	0.944	1	0.937	0.993	1
	MA-max	0.485	0.692	0.999	0.881	0.999	1	1	1	1	1	1	1	1	1	1
	RE-mean	0.046	0.083	0.238	0.180	0.336	0.715	0.398	0.604	0.936	0.567	0.830	0.993	0.767	0.950	1
	BD-mean	0.068	0.132	0.335	0.242	0.442	0.797	0.483	0.723	0.966	0.695	0.893	0.994	0.852	0.968	1
	GR-mean	0.460	0.705	0.962	0.835	0.956	1	0.953	1	1	1	1	1	1	1	1
	GA-mean	0.314	0.378	0.540	0.528	0.640	0.870	0.688	0.828	0.985	0.830	0.940	1	0.925	0.992	1
	MA-mean	0.474	0.681	0.996	0.868	0.996	1	0.995	1	1	1	1	1	1	1	1

**Fig 14:**
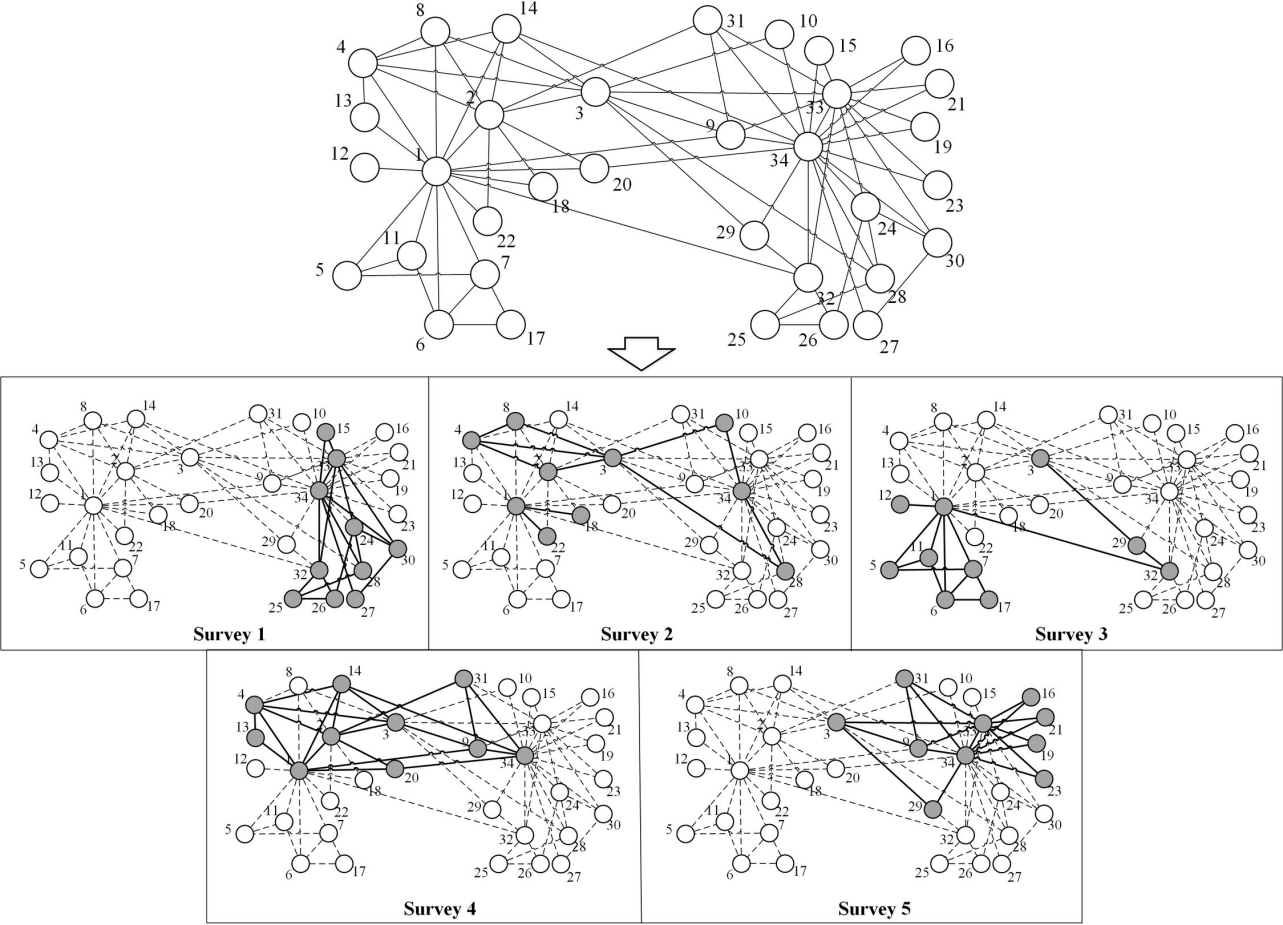
The sampling solution of Zachary’s Karate Club network with *k* = 0.3*N* and *m* = 5. The upper part is the topology of Zachary’s Karate Club network. The lower part is the solution of extracting multiple subgraphs. In the solution part, grey nodes represent included nodes and white nodes represent omitted nodes. The dashed lines represent the unobserved ties, while the bold solid lines represent the extracted ties.

By decoding the best chromosomes generated by the proposed algorithm, we can extract the specific sampling solution in each subgraph. Fig 14 presents one of the best extraction methods for Zachary’s Karate Club network with *k* = 0.3*N≈*10 and *m* = 5. The present solution is able to collect all the edges, i.e. the covering *C* = 100%. In Zachary’s Karate Club network, nodes 1, 2, 3, 33, 34 are key individuals who have the highest centrality, and we find that at least two central nodes are needed to include as many edges as possible. However, there is no solution that includes all five central nodes within the same subgraph. This is because a pair of central nodes may be disconnected, while including these nodes may not collect any edges. For example, investigating nodes 1, 3, 34 cannot collect any edges although they have important positions in the network. This suggests that including the central nodes is important, but extracting only the central nodes may not lead to a result that gives the best coverage.

**Fig 15:**
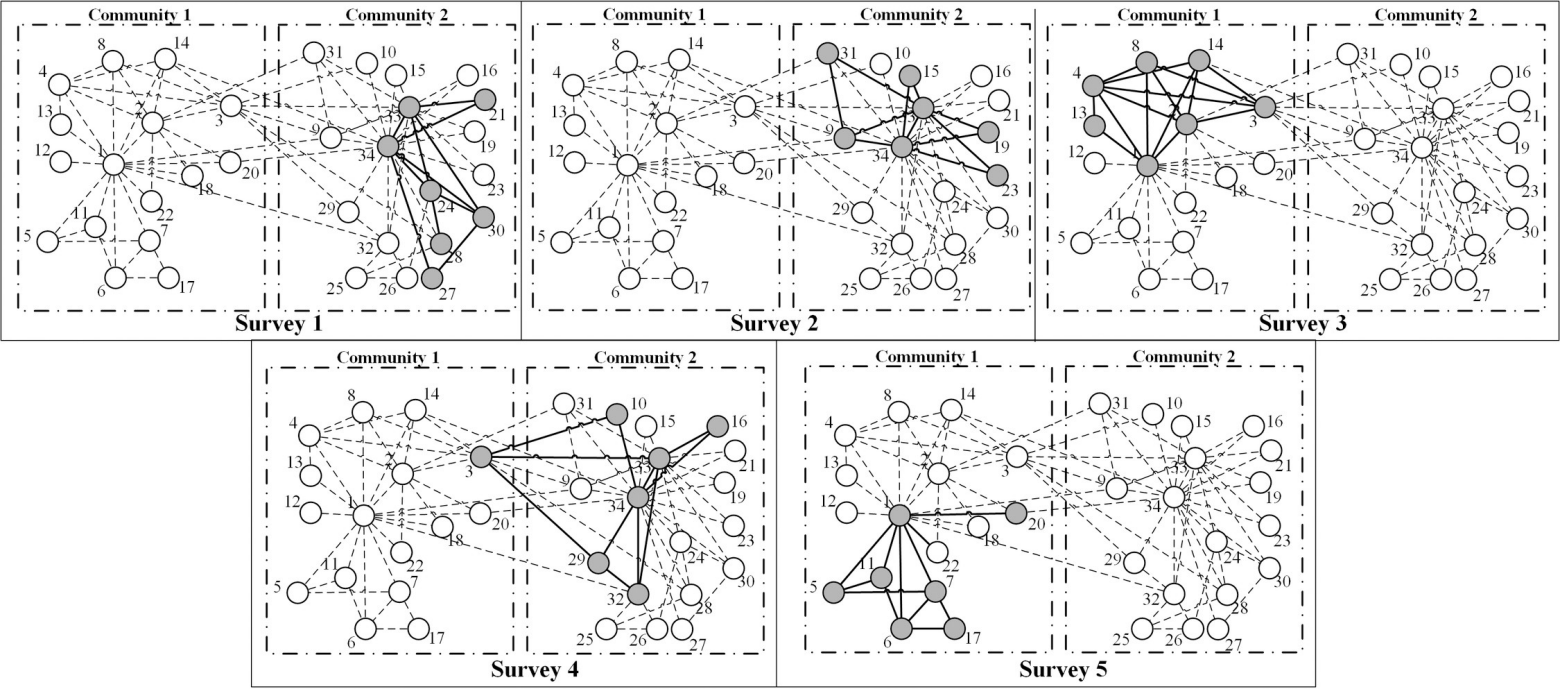
The sampling solution of Zachary’s Karate Club network with *k* = 0.2*N* and *m* = 5. The club members are divided into two communities. Included nodes are grey, and white nodes represent omitted nodes. The dashed line represents the unobserved ties, while the bold solid line represents the extracted ties.

We find that the optimal method is associated with the community structure. Zachary’s Karate Club network is a typical network with characteristic community structure [67]. The network can be naturally divided into two communities where edges are denser within the same community but sparser between the different communities. Fig 15 presents the optimized solution of Zachary’s Karate Club network with *k* = 0.2*N≈*7 and *m* = 5. This solution cannot collect all the edges (*C* = 0.769) because of the limited subgraph size. Most of the extracted nodes within the same communities are included in the one independent subgraph, which suggests that collecting nodes within the same community in each subgraph is helpful for collecting as many edges as possible; we call this the “community collecting method” (CCM). However, the edges between different communities can be hard to detect using CCM. Therefore, CCM is appropriate where the subgraph size or number are so limited that the optimized solution cannot collect all the edges (in other words, *C*<1). Another limitation of CCM is that it may not work effectively on networks without community structure (modularity *Q*<0.3) [7].

**Fig 16:**
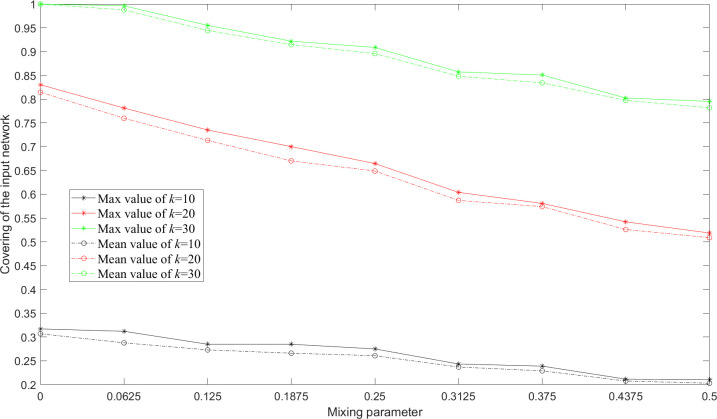
Covering of the benchmark networks for different mixing parameters given the number of subgraphs *m* = 10 and the subgraph size *k* = 10, 20 and 30 acquired by MA-M*m*C*k*SP.

In order to test the performance of CCM, we ran MA-M*m*C*k*SP on the benchmark networks proposed by Lancichinetti et al. [69]. Each network consists of 128 nodes with the average degree of 16. These nodes are evenly assigned one of the clustering attributes {1, 2, 3, 4}. We introduce a mixing parameter that denotes the fraction of edges for one node linking to other nodes with different clustering attributes. A higher mixing parameter represents a smaller modularity of the input network. We generated nine networks for values of mixing parameter ranging from 0 to 0.5. Fig 16 shows the covering result for different mixing parameters given the number of subgraphs *m* = 10 and the subgraph size *k* = 10, 20 and 30. We find that the extracted subgraphs are less covering as the mixing parameter increases. This is because the optimal solution is based on CCM, while CCM performs less efficiently when the feature of community structure of the input network declines.

## 5. Conclusion and discussion

The present study provides a new perspective on addressing the multiple densest subgraph problem. We advance research on this topic by formulating the problem of covering the input network as an optimization problem and propose a model that maximizes the covering of the observed network by extracting multiple subgraphs. A memetic algorithm combined with a genetic algorithm and local search optimizes the extraction in each independent subgraph.

The proposed algorithm can solve the optimization problem effectively. Compared to adding the number of extractions, increasing subgraph size is more helpful in improving the coverage of the network. Including nodes with higher centrality is necessary, but investigating only those nodes cannot fully reproduce the input network structure because the common edges connected with normal crowds (nodes with lower centrality) can easily be ignored. When subgraph size or numbers are constrained, the community collecting method, which includes nodes within the same community in each subgraph, can be an effective way of enhancing the covering. A suggestion for practitioners is to recognize the potential community structure of research objects before conducting the extractions.

**Fig 17:**
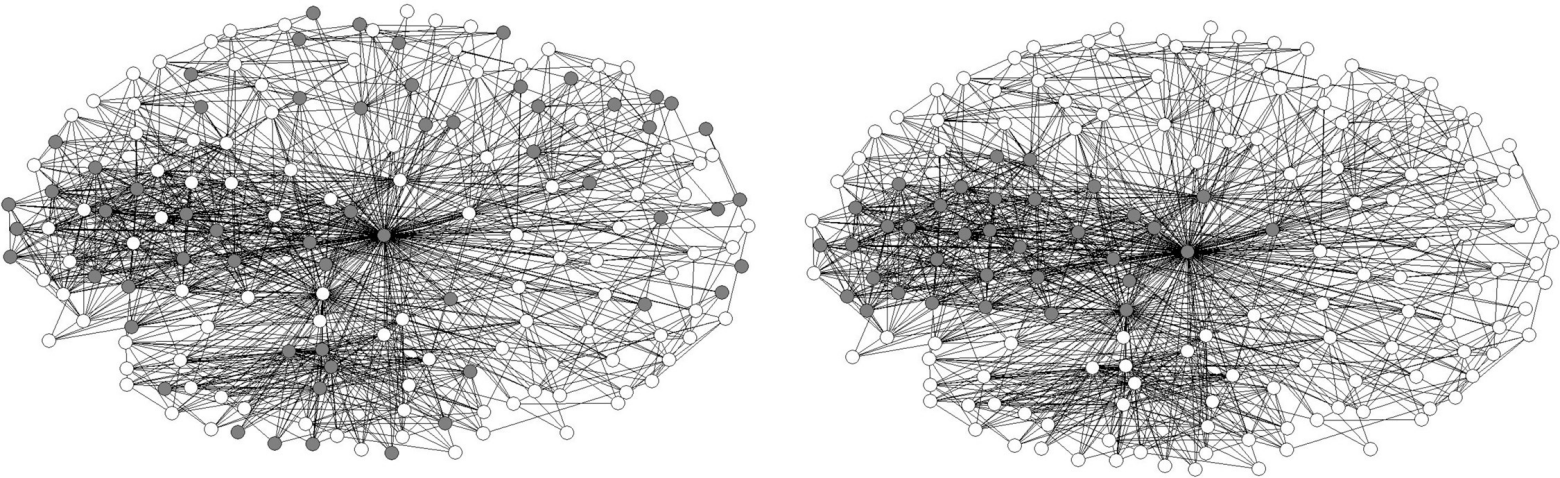
Identification of central and marginal members of the ADS migrant workers’ network using different methods. (a) uses the number of friends or acquaintances in the individual-level questionnaire; (b) uses the core-periphery model. The dark nodes are central members, while the white nodes are marginal members.

From a sociological review, previous research has highlighted the effectiveness of random sampling [27, 45, 57], but this method is not effective when surveys are conducted repeatedly. This is because random sampling in multiple surveys leads to redundancy, where an edge may be detected many times. The top-down sampling method (choosing the top nodes ordered by size) is also of limited value in repeated surveys, because edges connected by nodes with different rank sizes cannot be collected. Including central nodes helps to enhance the covering, but including only the representative nodes may not lead to a representative result. On the other hand, node size is difficult to estimate precisely in social networks. Before acquiring the whole structure of a network, it is difficult to judge whether an individual is a central or marginal member. An illustration is presented in Fig 14, which shows the difference between different methods in recognizing core nodes. The network in Fig 17 is the ADS migrant workers’ network. By asking “how many friends or acquaintances do you have in Shenzhen (ADS is located in this city)?” in the individual-level questionnaire, we can divide the company members into “big-size” individuals, who have 30 or more friends or acquaintances, and small-size individuals, who do not have as many as 30 friends (see Fig 17A). By applying the core-periphery model [5] to the whole network, we can also find big-size individuals and small-size individuals as shown in Fig 17B. This figure is derived using Eq (5), where *α*_*ij*_ is relationship between nodes *i* and *j*, and *c*_*i*_ is one of node *i*’s attributes (core or periphery), “●” indicates a missing value which treat the off-diagonal regions of *α*_*ij*_ as missing data that helps maximize density in the core and minimize density in the periphery. The inconsistency between (a) and (b) suggests that top-down sampling may choose some fake big-size nodes which undermines the accuracy of network estimation.


$$\text{Maximize}\rho =\sum_{i,j}\alpha_{ij}\delta_{ij},\;\delta_{ij}=\left\{\begin{array}{l} 1,\;\text{if}\;c_{i}=\text{core}\;\text{and}\;c_{j}=\text{core}\\ 0,\;\text{if}\;c_{i}=\text{periphery}\;\text{and}\;c_{j}=\text{periphery}\\ •,\;\text{otherwise} \end{array}\right.$$ (5)

In most natural settings, practitioners have no idea as to the real structure of the actual network. In order to collect all the potential edges, practitioners should assume that the actual network is completely connected, so that each pair of nodes is connected. The proposed algorithm can also be applied to the covering for a completely connected network. Despite the merits of these new proposals, there are some limitations to the present study. The algorithm may sometimes be trapped in a local maximum, and we plan to design a more intelligent algorithm in the future. The objective function (coverage of the input network) in this paper is the detected number of edges divided by the total number of edges, while other indices, such as centrality, might also be employed in the optimization model. A meaningful analysis of social networks requires both individual-level and network-level investigations, and thus an index for measuring the covering of multiple subgraphs that considers both individual and relational attributes needs to be designed in the future.
